# Dr. James Nevins Hyde

**Published:** 1910-10

**Authors:** 


Dr. James Nevins Hyde, of Chicago, died suddenly at his summer home at Trouts Neck, Me., September 6, 1910, aged 70 years. Dr. Hyde, received his early education at Andover, Mass., and then was graduated from Yale University and the medical school at the University of Pennsylvania. He was an assistant surgeon in the United States navy during the Civil War. At the close of the war he resigned from the service to enter private practice, becoming one of Chicago's foremost physicians, being especially eminent in dermatology. He was connected at various times with the medical faculties of the Northwestern University and the University of Chicago, and with the faculty of Rush Medical College. Dr. Hyde was the author of a treatise on diseases of the skin that has passed through many editions, and which is quoted the world over as an authoritative textbook.

